# Co-morditities of environmental diseases: A common cause

**DOI:** 10.2478/intox-2014-0016

**Published:** 2014-12-30

**Authors:** Harold I. Zeliger

**Affiliations:** Zeliger Research, South Portland, ME, USA

**Keywords:** environmental disease, co-morbidity, diabetes, cardiovascular disease, neurological disease

## Abstract

The global pandemic of non-vector borne environmental diseases may, in large part, be attributed to chronic exposures to ever increasing levels of exogenous lipophilic chemicals. These chemicals include persistent organic pollutants, semi-volatile compounds and low molecular weight hydrocarbons. Such chemicals facilitate the sequential absorption of otherwise not absorbed more toxic hydrophilic species that attack numerous body organs and systems, leading to environmental disease. Co-morbidities of non-communicable environmental diseases are alarmingly high, with as many as half of all individuals chronically ill with two or more diseases. Co-morbidity is to be anticipated, since all of the causative chemicals identified have independently been shown to trigger the individual diseases.

## Introduction

The prevalence of non-vector borne environmental disease in the world has reached pandemic proportions (Murray *et al.*, 2012). In the United States alone, half of all adults have at least one environmental disease and more than a quarter of the adult population suffers from two or more co-morbid environmental diseases (Bauer *et al.*, 2014; Jakovljevic *et al.*, 2013; van Oostrom *et al.*, 2012). Indeed, rapid increases in incidences of environmental diseases have not been limited to the industrialized areas of the globe but have also spread to remote areas including those inhabited by indigenous populations in the tropics and near the poles (Vos *et al.*, 2012). The rapid rise in the prevalence of these diseases can only be linked to environmental effects (Boyle *et al.*, 2010). A common thread in many environmental diseases is the presence of exogenous lipophilic toxic chemicals in the bodies of those affected (Zeliger, 2013; Zeliger, 2013a; Zeliger 2013b).

It has been previously reported that chemically sensitive individuals exposed to low molecular weight hydrocarbons (LMWHCs) had numerous co-morbidities (Zeliger *et al.* 2012). It can now be reported that exposures to all exogenous lipophilic chemicals cause co-morbidities and that co-morbidity of environmental disease is not limited to just the chemically sensitive people. Exposure to and retention of lipophilic persistent organic pollutants (POPs) semi-volatile and volatile exogenous lipophilic chemicals has been associated with increased prevalence of type 2 diabetes (T2D) (Carpenter, 2008; Lee *et al.*, 2010; Zeliger 2013), cardiovascular disease (Zeliger, 2013a), and neurological disease (Zeliger 2013b). Many other environmental diseases, that affect virtually all body systems, are also associated with exposure to and retention of exogenous lipophilic chemical species. These include: immunological (Marie *et al.*, 2013), musculoskeletal (Al-Bashri *et al.*, 2013; Struijs *et al.*, 2006); and respiratory diseases(Cazzola *et al.*, 2013; Varela *et al.*, 2013; Molen, 2010); as well as numerous cancers (Habib *et al.*, 2013; Sorensen, 2013; van Baal *et al.,* 2010).

A unifying explanation for induction of environmental disease by absorbed exogenous lipophilic chemicals has been previously presented (Zeliger, 2013). Review of the medical and toxicological literature shows that the onset of these diseases is associated with the accumulation of exogenous lipophilic chemicals in body serum, (Gallo *et al.*, 2011; Lee *et al.*, 2011; Lee *et al.*, 2007; Philibert *et al.*, 2009; Cortu *et al.*, 2007). A dose dependent relationship between POPs serum levels and type 2 diabetes (T2D), for example, has been shown to exist (Cortu *et al.*, 2007; Lee *et al.*, 2006). Lipophilic cell membranes are not permeable to most hydrophilic chemicals. Lipophilic chemicals act as solvents and carriers for impermeable hydrophiles to facilitate absorption of species which would not otherwise permeate through the cells’ lipophilic barriers (Zeliger 2003).

It has also been previously shown that mixtures of toxic lipophilic and hydrophilic species produce enhanced toxicities, low-level effects and attacks on organs and/or systems not known to be impacted by either species alone (Zeliger, 2003; Zeliger, 2011). Such effects have been observed following simultaneous exposures to mixtures of lipophililc and hydrophilic chemicals. Environmental disease can be triggered by the initial absorption and retention of lipophilic species followed by the sequential uptake of hydrophilic species that then act together as a toxic mixture, with the absorption of different hydrophiles accounting for the onset of different diseases (Zeliger *et al.*, 2012; Zeliger, 2013; Zeliger, 2013a; Zeliger 2013b).

Though different diseases involve attacks on widely disparate organs and systems, co-morbidity rates are high when individuals are exposed to environmental lipophilic toxins (Zeliger *et al.*, 2012). The onsets of co-morbid diseases do not follow set patterns. Published studies show that individuals with two co-morbid diseases, *e.g.*, T2D and hypertension, are just as likely to become ill with one first as the other first (Sowers *et al.*, 2001), for example. The wide prevalence of co-morbid environmental diseases and the lack of a pattern of onset strongly suggests the common cause for these diseases that has been previously reported on (Zeliger, 2013; Zeliger, 2013a; Zeliger, 2013b).

## Methods

The results presented here are based upon a literature review of numerous studies on the toxic effects of the chemicals on the body. These studies include epidemiological and case studies. Adverse effects on health were in all instances diagnosed by appropriate clinical examinations. Data for pairs of co-morbidities were carried out by literature searches for the words, “co-morbidity” and the names of the two diseases, “cardiovascular disease” and “musculoskeletal disease”, for example.

## Results

Specific lipophilic chemicals associated with multiple environmental diseases include those previously reported to be associated with T2D, cardiovascular disease and neurological disease. These include non-volatile POPs, semi-volatile and volatile species (Zeliger, 2013; Zeliger, 2013a; Zeliger, 2013b).

Major environmental diseases that have been associated with lipophilic exposure include immunological, neurological, neurodegenerative reproductive, cardiovascular, metabolic, musculoskeletal and carcinogenic ones. These are listed in Table 1.

**Table 1:** Major diseases associated with exposures to lipophilic environmental chemicals.

Type 2 diabetes (T2D): Including metabolic syndrome.
Cardiovascular (CVD): Including atherosclerosis, myocardial infarction, hypertension, stroke, coronary heart disease, peripheral heart disease, ischemic heart disease and cardiac autonomic function.
Neurological (NRD): Including central nervous system disorders (cognitive, motor and sensory), and peripheral nervous system disorders (neuropathies).
Neurodevelopmental (NDV) including autism and attention deficit/hyperactivity disorder (ADHD).
Neurodegenerative (NDG) including Alzheimer's disease, Parkinson's disease and amyotrophic lateral sclerosis (ALS).
Musculoskeletal (MKS) including osteoarthritis and fibromyalgia (FM).
Immunological (IMM) including allergic reaction, autoimmune diseases and chronic fatigue syndrome (CFS).
Respiratory (RES) including asthma and chronic obstructive pulmonary disease (COPD).
Chemical sensitivity (CMS) including hypersensitivity to inhaled and dermal contact moieties.
Obesity (OBS).
Cancers (CAN) in multiple organs.

**Figure 1:**
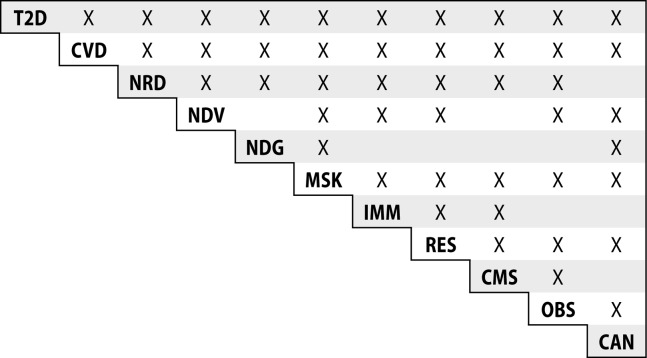
Co-morbidities of chemically induced environmental diseases. References for co-morbidity disease pairs are contained in Table 2. X denotes the existence of co-morbidity between the two diseases.

All of the diseases listed in Table 1 are co-morbid with other environmental diseases that are known to be triggered by exposures to lipophilic chemicals. Figure 1 shows the co-morbidities of the 11 types of these diseases with each other. Table 2 lists co-morbid disease pairs and the references for these. It is of note that of the 55 binary combinations possible, 45 (82%)% have been shown to be co-morbid to date.

**Table 2:** References for environmental disease co-morbidities.

Disease Pair	References
T2D - CVD	1995; Colosia *et al.*, 2013; Mannino *et al.*, 2008; Struijs *et al.*, 2006; Sowers *et al.*, 2001; Ramakrishnan *et al*., 2013.
T2D - NRD	Blackman *et al.*, 2013; Sowers *et al.*, 1995; Uzun *et al.*, 2009; Katon, 2008; Struijs *et al.*, 2006; Lee *et al.*, 2008.
T2D - NDV	Kohane *et al.*, 2012.
T2D - NDG	Bannon, 2002; Duthie *et al.*, 2011; Gage *et al.*, 2003; Struijs *et al.*, 2006.
T2D - MSK	Al-Bishri *et al.*, 2013; Mannino *et al.*, 2008; Slater *et al.*, 2011.
T2D - IMM	2009.
T2D - RES	van der Molen, 2010; Varela *et al.*, 2013; Cazzola *et al.*, 2013; Mannino *et al.*, 2008; Struijs *et al.*, 2006; Chatila *et al.*, 2008.
T2D - CMS	Zeliger *et al.*, 2012.
T2D - OBS	Colosia *et al.*, 2013; Sowers *et al.*, 2001; Khaodhiar *et al.*, 1999.
T2D - CAN	Habib *et al.*, 2013; van Baal *et al.*, 2011.
CVD - NRD	2008; Uzun *et al.*, 2009; Larsen *et al.*, 2009.
CVD - NDV	Tyler *et al.*, 2011.
CVD - NDG	Armon, 2004; Bannon, 2011; Duthie *et al.*, 2011; Zamrini *et al.*, 2004; Gage *et al.*, 2003; Perju-Dumbrava *et al.*, 2014.
CVD - MSK	Al-Bishri *et al.*, 2013; Slater *et al.*, 2011; Hudon *et al.*, 2008.
CVD - IMM	Marrie et al, 2013; Aaron *et al.*, 2000.
CVD - RES	Karlstad *et al.*, 2012; van der Molen, 2010; Varela *et al.*, 2013; Chatila *et al.*, 2008.
CVD - CMS	Zeliger *et al.*, 2012.
CVD - OBS	2001; Khaodhiar *et al.*, 1999.
CVD - CAN	Kreatsoulas *et al.*, 2014; Sorensen, 2013.
NRD - NDV	Cristino *et al.*, 2013; Simonoff *et al.*, 2008; Jensen *et al.*, 2014.
NRD - NDG	Varela *et al.*, 2013; Gage *et al.*, 2003.
NRD - MSK	Marrie *et al.*, 2013; Blackman *et al.*, 2013; McIntyre *et al.*, 2008; Hudon *et al.*, 2008.
NRD - IMM	Marrie *et al.*, 2013; Aaron *et al.*, 2001.
NRD - RES	Marrie *et al.*, 2013; Karlstad *et al.*, 2012; Blackman *et al.*, 2013; van der Molen, 2010; McIntyre *et al.*, 2008; Cazzola *et al.*, 2013; Chatila *et al.*, 2008.
NRD - CMS	Zeliger *et al.*, 2012
NRD - OBS	2008; Khaodhiar *et al.*, 1999; Luppino *et al.*, 2010.
NDV - RES	Fasmer *et al.*, 2011.
NDV - OBS	Suren *et al.*, 2014.
NDV - CAN	Crespi, 2011.
NDG - MSK	2003.
NDG - CAN	Crespi, 2011; Zamrini *et al.*, 2004; Gage *et al.*, 2003
MSK - IMM	2009; Ciccone *et al.*, 2003.
MSK - RES	van der Molen, 2010; Slater *et al.*, 2011; Chatila *et al.*, 2008; Hudon *et al.*, 2008.
MSK - CMS	Zeliger *et al.*, 2012
MSK - OBS	1999; Hudon *et al.*, 2008;
MSK - CAN	Sorensen, 2013.
IMM - RES	Karlstad *et al.*, 2012; Pinart *et al.*, 2014.
IMM - CMS	Zeliger *et al.*, 2012; Ziem *et al.*, 1995; Jason *et al.*, 2000.
RES - CMS	Zeliger *et al.*, 2012; Caress *et al.*, 2005.
RES - OBS	van der Molen, 2010; Cazzolam *et al.*, 2013; Jung *et al.*, 2014.
RES - CAN	Varela *et al.*, 2013; Sorensen, 2013.
CMS - OBS	Zeliger *et al.*, 2012.
CMA - CAN	Zeliger *et al.*, 2012
OBS - CAN	Khaodhiar *et al.*, 1999.

**Abbreviations:** T2D - type 2 diabetes; CVD - cardiovascular disease; NRD - neurological disease; NDV - neurodevelopmental disease; NDG - neurodegenerative disease; MSK - musculoskeletal disease; IMM - immunological disease; RES - respiratory disease; CMS - chemical sensitivity; OBS - obesity; CAN - cancer

## Discussion

The environmental diseases reported here are late onset diseases that generally follow decades of living during which physiological breakdown of many systems occur (Wright *et al.*, 2002). All are triggered by a combination of genetic susceptibility and environmental exposure (Zhang *et al.*, 2010). Several mechanisms have been proposed the account for such breakdowns. These include: oxidative stress (Uttara *et al.*, 2009; Bolanos *et al.*, 2009); epigenetic effects (Jakovcevski *et al.*, 2012; Urdinguio *et al.*, 2009; Baccarelli *et al.*, 2009); low intensity inflammation (Miller *et al.*, 2008; Leonhard *et al.*, 2006;); and endocrine disruption (Weiss, 2012; Mostafalou *et al.*, 2013; Colborn *et al.*, 1997). One theory of co-morbidities of environmental diseases is that there are phenotype connections between diseases, *i.e.*, that patients are affected by diseases that are connected to other diseases by a Phenotype Disease Network (Hidalgo *et al.*, 2009; Zhang *et al.*, 2010; Lee *et al.*, 2008). All of these theories are consistent with what is reported here, since exposures to all the lipophilic chemical types described above (POPs, semi-volatile organic compounds, and volatile organic compounds) have been independently been associated with each of the diseases listed in Table 1 (Zeliger, 2013; Zeliger, 2013a; Zeliger, 2013b). It is beyond the scope of this paper to examine these mechanisms in detail. Readers are directed to the references cited for elaboration.

Since all of the diseases listed in Table 1 have been related to exogenous lipophilic adsorption (Zeliger, 2013; Zeliger 2013a; Zeliger 2013b) it is to a great extent predictive that individuals ill with one of these diseases will be co-morbidly ailing with at least one other of these diseases (Zeliger *et al.*, 2012). This can be stated emphatically, as there are numerous studies in the literature showing, that where individuals are co-morbid with two of these diseases, the co-morbidities are independent of the order of onset of the two diseases, *i.e.*, that either of the diseases can precede the other. The following serve as examples of these studies. Somers *et al.* reported that individuals with autoimmune disease show higher than expected co-morbidities with musculoskeletal disease and type 2 diabetes and that in both instances either of the diseases could precede the other (Somers *et al.*, 2009). In people co-morbid with metabolic syndrome and mental health disorders, either condition can precede the other (Nousen *et al.*, 2013). Obesity and depression are common co-morbid conditions and either one can precede the other (Luppino *et al.*, 2012). Hypertension is about twice as common in diabetics as in those without diabetes and either disease can precede the other (Sowers *et al.*, 1995; Sowers *et al.*, 2001).

Based on the above, it can be stated that one cause of numerous environmental diseases and co-morbidities is chronic lipophilic exposure to lipophiles such as persistent organic pollutants (POPs), semi-volatile exogenous chemicals (SVOCs) and low molecular weight hydrocarbons (LMWHCs). Examples of POPs are polychlorinated biphenyls (PCBs) and organochlorine pesticides (OCs). Examples of semi-volatile compounds are bisphenol A, phthalates and polynuclear aromatic hydrocarbons. Examples of volatile organic compounds are 8 carbon or less aliphatic and single-chain aromatic hydrocarbons. POPs are slowly metabolized and eliminated and are stored in white adipose tissue, from where they are slowly released into the blood stream. SVOCs are more rapidly metabolized and eliminated andLMWHCs are very rapidly metabolized and eliminated, but continued exposure to SVOCs and LMWHCs leads to continuous levels in the blood as well. A steady state of lipophilic load is in effect in the body, and since lipophiles facilitate the absorption of hydrophiles, a body containing high levels of lipophiles is more likely to absorb toxic levels of hydrophiles when exposure to these occurs than one with low levels of lipophiles. This is shown by the dose-response relationships for the onset of T2D, cardiovascular disease and neurological disease (Cortu *et al.*, 2007; Lee *et al.*, 2006; Zeliger, 2013a; Zeliger, 2013b). As a wide variety of exogenous lipophiles have been shown to cause all of the diseases in Table 1, it is total exogenous lipophilic load, regardless of chemical species, that is more predictive of the onset of disease than single chemical considerations.

Further credence to the theory just proposed is provided by the following considerations:

1. Not only do exogenous lipophiles cause these diseases, but one of these diseases, obesity has also been shown to cause the absorption of lipophiles (See below).
2. One in four individuals with one of these diseases is likely to be stricken with at lease one more of these diseases (Bauer *et al.*, 2014; Jakovljevic *et al.*, 2013; van Oostrom *et al.*, 2012).
3. Eleven (11) types of environmental diseases are listed in Table 1. Of the 55 binary combinations of diseases possible, 45 (82%), have been shown to be co-morbid (see Figure 1).
4. All the diseases in Table 1 are late-onset ones, coming mostly after decades of exposures (Fortin *et al.*, 2005).

A consideration of obesity is in order here. Body Mass Index (BMI) of 30 or greater is considered obese (Luppino *et al.*, 2010). BMI is a predictor of human adipose tissue concentration of POPs (Vaclavik *et al.*, 2006). This is consistent with the fact that obesity is usually associated with CVS, T2D and other diseases, as adipose tissue releases the lipophiles it holds to the blood stream. Obesity is itself caused by POPs, phthalates, bisphenol A and other exogenous lipophiles (Dirinick *et al.*, 2014; Choi *et al.*, 2014; Langer *et al.*, 2014; Lee *et al.*, 2014; Simmons *et al.*, 2014; Lee *et al.*, 2011a). Being obese and having high serum endogenous lipophiles (cholesterol and tryglycerides) contributes to the absorption of these exogenous lipophiles (Wang *et al.*, 2007; Vaclavik *et al.*, 2006). This sets up what is termed here as the Obesity (OBS) – Lipophile (LIP) – Disease (DIS) triangle:

**Figure:**
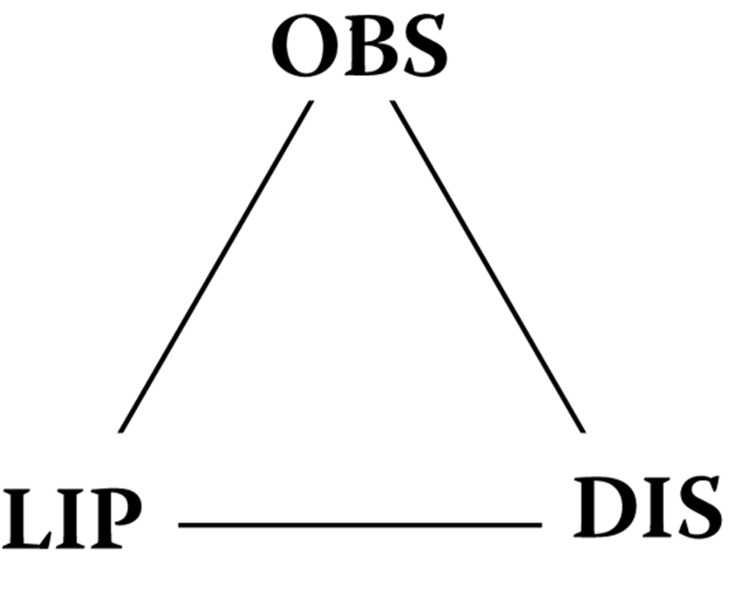


Obesity causes the absorption of toxic lipophiles which in turn cause disease. Toxic lipophiles cause obesity which in turn causes the further absorption of lipophiles which cause disease. Disease causes obesity which causes the absorption of lipohiles which in turn causes further disease. (Dirinick *et al.*, 2014; Choi *et al.*, 2014; Langer *et al.*, 2014; Lee *et al.*, 2014; Simmons *et al.*, 2014).

Finally, it is not implied that lipophilic exposure is the only cause for environmental disease. For example, heavy metals (including arsenic, cadmium, chromium, cobalt, copper, mercury and nickel) are known trigger environmental diseases, including type 2 diabetes, cardovascular diseases and neurological diseases (Carocci *et al.*, 2014; Caciari *et al.*, 2013; Kuo *et al.*, 2013; Baccarelli *et al.*, 2009; Khan, et al., 2014; Agarwal *et al.*, 2011; Mates *et al.*, 2010).

## Conclusions

In conclusion, it has been previously shown that chemically sensitive individuals had numerous co-morbidities when exposed to LMWHCs (Zeliger *et al.*, 2012). It can now be stated that exposures to all exogenous lipophiles (POPs and SVOCs, as well as LMWHCs) also produce co-morbidities of environmental diseases in all segments of the population. Exposures to lipophiles result in numerous co-morbid disease pairs affecting widely differing organs and systems. It is theorized that all chronic exposures to lipophilic exogenous chemicals lead to steady states of such compounds in human blood and can cause of a wide range of environmental diseases that affect numerous body organs and systems. Since the lipophiles serve are carriers for the sequential absorption of more toxic hydrophiles, disease onset is dictated not by the individual chemistries of the lipophiles, but by total lipophilic load in the blood. Lipophilic exposure promotes obesity, which promotes the absorption of additional exogenous lipophiles that promote further environmental disease. An obesity-lipophile-disease triangle which promotes the furthering of environmental disease is thus defined.
